# Molecular Pathways Mediating Immunosuppression in Response to Prolonged Intensive Physical Training, Low-Energy Availability, and Intensive Weight Loss

**DOI:** 10.3389/fimmu.2019.00907

**Published:** 2019-05-03

**Authors:** Heikki V. Sarin, Ivan Gudelj, Jarno Honkanen, Johanna K. Ihalainen, Arja Vuorela, Joseph H. Lee, Zhenzhen Jin, Joseph D. Terwilliger, Ville Isola, Juha P. Ahtiainen, Keijo Häkkinen, Julija Jurić, Gordan Lauc, Kati Kristiansson, Juha J. Hulmi, Markus Perola

**Affiliations:** ^1^Genomics and Biomarkers Unit, National Institute for Health and Welfare, Helsinki, Finland; ^2^Research Program for Clinical and Molecular Metabolism, Faculty of Medicine, University of Helsinki, Helsinki, Finland; ^3^Genos Glycoscience Research Laboratory, Zagreb, Croatia; ^4^Pedia Laboratory, Clinicum, University of Helsinki, Helsinki, Finland; ^5^Faculty of Sport and Health Sciences, Neuromuscular Research Center, Biology of Physical Activity, University of Jyväskylä, Jyväskylä, Finland; ^6^Department of Health Sciences, Swedish Winter Sports Research Centre, Mid Sweden University, Östersund, Sweden; ^7^Sergievsky Center, Taub Institute and Departments of Epidemiology and Neurology, Columbia University, New York, NY, United States; ^8^Department of Biostatistics, Columbia University, New York, NY, United States; ^9^Division of Medical Genetics, Departments of Psychiatry, Genetics & Development, Sergievsky Center, New York State Psychiatric Institute, Columbia University, New York, NY, United States; ^10^Department of Biochemistry and Molecular Biology, Faculty of Pharmacy and Biochemistry, University of Zagreb, Zagreb, Croatia; ^11^Department of Physiology, Faculty of Medicine, University of Helsinki, Helsinki, Finland

**Keywords:** immunosuppression, low energy availability, physical training, bioinformatics, weight loss

## Abstract

Exercise and exercise-induced weight loss have a beneficial effect on overall health, including positive effects on molecular pathways associated with immune function, especially in overweight individuals. The main aim of our study was to assess how energy deprivation (i.e., “semi-starvation”) leading to substantial fat mass loss affects the immune system and immunosuppression in previously normal weight individuals. Thus, to address this hypothesis, we applied a high-throughput systems biology approach to better characterize potential key pathways associated with immune system modulation during intensive weight loss and subsequent weight regain. We examined 42 healthy female physique athletes (age 27.5 ± 4.0 years, body mass index 23.4 ± 1.7 kg/m^2^) volunteered into either a diet group (*n* = 25) or a control group (*n* = 17). For the diet group, the energy intake was reduced and exercise levels were increased to induce loss of fat mass that was subsequently regained during a recovery period. The control group was instructed to maintain their typical lifestyle, exercise levels, and energy intake at a constant level. For quantification of systems biology markers, fasting blood samples were drawn at three time points: baseline (**PRE**), at the end of the weight loss period (**MID** 21.1 ± 3.1 weeks after **PRE**), and at the end of the weight regain period (**POST** 18.4 ± 2.9 weeks after **MID**). In contrast to the control group, the diet group showed significant (false discovery rate <0.05) alteration of all measured immune function parameters—white blood cells (WBCs), immunoglobulin G glycome, leukocyte transcriptome, and cytokine profile. Integrative omics suggested effects on multiple levels of immune system as dysregulated hematopoiesis, suppressed immune cell proliferation, attenuated systemic inflammation, and loss of immune cell function by reduced antibody and chemokine secretion was implied after intense weight loss. During the weight regain period, the majority of the measured immune system parameters returned back to the baseline. In summary, this study elucidated a number of molecular pathways presumably explaining immunosuppression in individuals going through prolonged periods of intense training with low-energy availability. Our findings also reinforce the perception that the way in which weight loss is achieved (i.e., dietary restriction, exercise, or both) has a distinct effect on how the immune system is modulated.

## Introduction

Obesity and weight gain are associated with immune system dysfunction including impaired cell-mediated response, an increase in leukocyte counts, and induced low-grade systemic inflammation (1–5). Especially in overweight individuals, it has been demonstrated that exercise and exercise-induced weight loss alter overall health in numerous beneficial ways such as having positive effects on immune function related molecular pathways (5–7). Despite the undisputed evidence of the positive health benefits of weight loss with exercise and reduced energy intake in overweight individuals, the effect of intentional weight loss beyond normal levels of fat mass on immune system modulation related omics is still unknown. Some studies have suggested that rigorous prolonged exercise training combined with low-energy availability (i.e., undernutrition) in normal weight individuals may suppress immune system function (8–11).

Prolonged periods of intense exercise and energy deficit leading to weight loss are common in many sports, especially in aesthetic sports such as fitness and physique sports (12). These individuals, after years of training with *ad libitum* energy intake, go through intensive weight reduction periods (>10 weeks) preceding competitions to improve their muscular definition and aesthetic appearance by reducing body fat mass. Intensive weight reduction is typically accomplished by an exceptionally high volume of both resistance and endurance training and a low-energy intake (12). In these situations, alterations in immune function have also been suggested, but not thoroughly studied (13, 14).

To date, little is known about how prolonged intensive exercise training induced weight loss and altered adiposity modulates immune function, and only a few studies have applied multiple omics approaches to explain these complex system biological relations (15–18).

In the present study, we aimed to further elucidate potential biological mechanisms underlying weight loss induced modulation of immunity in order to uncover mediators of immunosuppression. This was done by studying and incorporating data from the leukocyte transcriptome, immunoglobulin G (IgG) glycome, along with white blood cell (WBC) distribution, and cytokine/chemokine profile in normal weight female individuals, before (**PRE**) and after long-term (>15 weeks) intensive training and low-energy availability leading to intensive weight loss (**MID**) and then again following the subsequent voluntary weight regain (**POST**). In general, we focus our discussion on hematopoiesis, WBC proliferation and responses, along with associated antibody and cytokine/chemokine mediated signaling in reference to adaptive and innate immune functions.

## Results

### Overview of the Study

**Figure 1:**
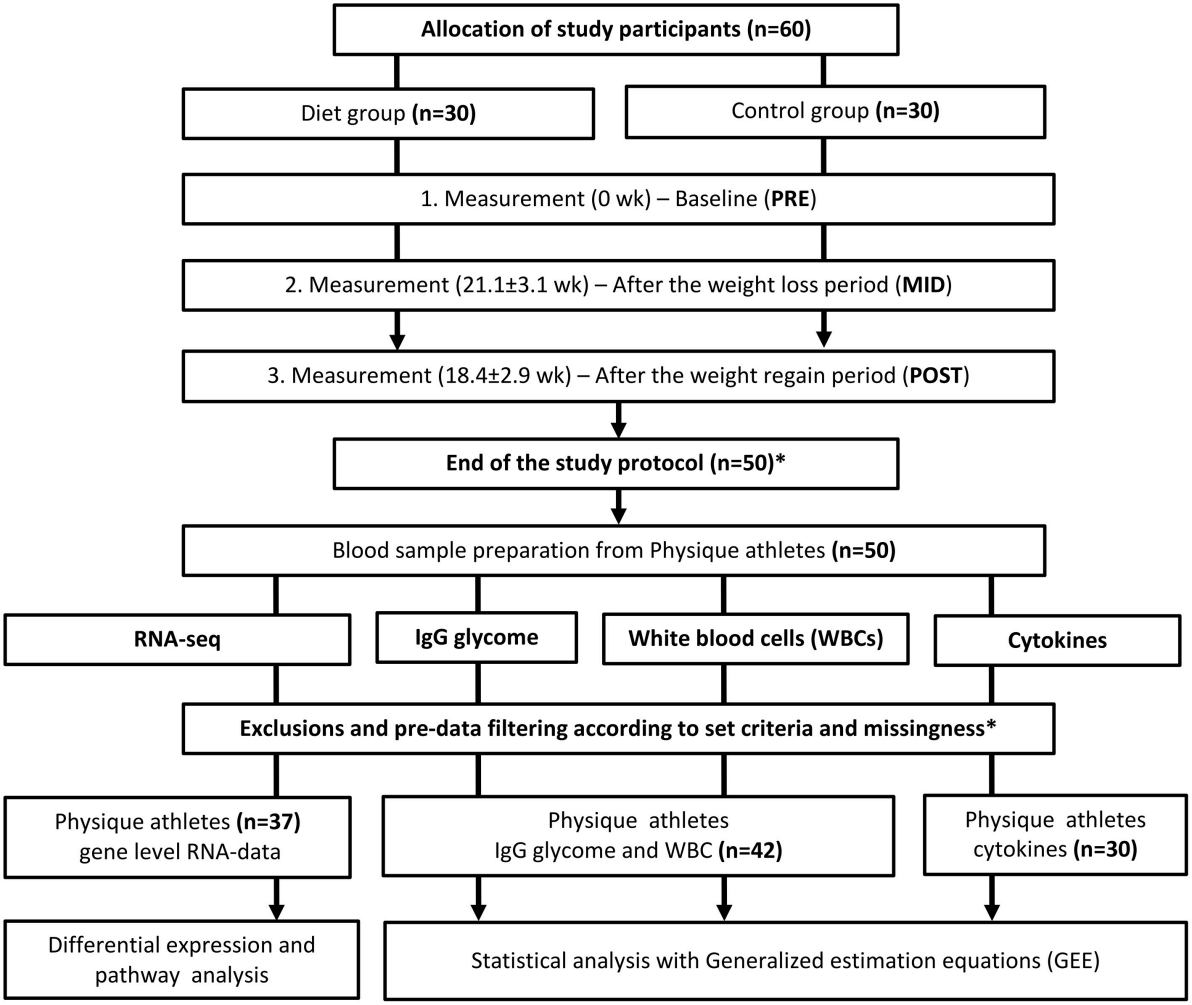
Study design and workflow. Study design and workflow are represented in a flowchart to illustrate the whole study protocol used. ^*^Of the 60 participants who started the study, 10 failed to complete the study regimen in a required manner. One control did not arrive for baseline testing (**PRE**) and the remaining nine participants (three from the diet group and six controls) were excluded out of the study because of a short duration of the weight regain period compared with the other participants or failure to completely follow the study instructions. Additional participants that lacked complete dietary records (*n* = 8) were excluded from the current omics study. Due to the high cost of large-scale data-set quantification, only individuals with minimal missing information were included in the study sample. After data quality control, also additional individuals were excluded from the analysis. The final number of participants used for the analysis on each data set is depicted in the figure. In total, we included samples from 42 participants (diet group *n* = 25, control group *n* = 17) in the bioinformatic analysis after the relevant exclusions. For the cytokine profile quantification, samples from only 30 individuals were analyzed due to high-cost of the analysis panel. Furthermore, sample size varied slightly between different downstream analyses due to incompleteness of omics or phenotype data.

In a sample of young (age 27.5 ± 4.0 years) previously normal weight (body mass index, BMI 23.4 ± 1.7 kg/m^2^) female physique athletes, we investigated immune function targeted multi-omics modulation at three time points: at baseline (**PRE**), at the end of the weight loss period (**MID** 21.1 ± 3.1 weeks after **PRE**), and at the end of the weight regain period (**POST** 18.4 ± 2.9 weeks after **MID**) (*n* = 25), and compared them with non-dieting controls (*n* = 17). An immune system function targeted systems biology approach included leukocyte derived RNA expression levels, IgG glycome, WBC count distribution, and cytokine/chemokine profile investigated in a longitudinal study setting including three time-point measurements (**PRE**, **MID**, and **POST**) (Figure 1). A detailed description of the study participants and design is provided in the Methods section.

### Physique Competition Diet Resulted in Distinct Alteration of Body Composition

The weight loss period (**PRE–MID**) resulted in a distinct (*P* < 0.05) reduction in body weight (~13%) and total body fat mass (~51%) in the diet group, as reported previously (12, 19) (Table 1). Weight loss in the study participants was accomplished by means of decreased energy intake (~18%) and increased total amount (metabolic equivalent hours per week, METh/wk) of exercise (~15%), resulting in decreased energy availability (~29%) (Table 1). Subsequently, the weight regain period (**MID–POST**) with decreased levels of exercise and increased energy intake reverted most of the observed anthropometric changes back to baseline levels in the diet group (Table 1) (12). In the control group, no significant (*P* > 0.05) changes were observed in anthropometric measurements throughout the study period (**PRE–POST**) (Table 1).

**Table 1:** Characteristics of body composition, exercise level, and energy intake alterations in the physique study groups.

**Measures**	**Group**	**Baseline (PRE)**	**After weight loss period (MID)**	**After weight regain period (POST)**
Weight (kg)	Diet	64.72 (6.92)	56.62 (5.51)^*^	63.17 (6.92)^*^
	Control	63.71 (5.07)	64.02 (5.76)	63.64 (5.55)
BMI (kg/m^2^)	Diet	23.54 (1.82)	20.6 (1.42)^*^	22.99 (2.03)^*^
	Control	23.08 (1.36)	23.2 (1.78)	23.05 (1.57)
Fat mass (kg)	Diet	14.88 (4.47)	7.17 (2.69)^*^	12.99 (4.21)^*^
	Control	14.19 (3.05)	14.87 (3.48)	14.39 (3.17)
Lean mass (kg)	Diet	47.69 (4.2)	48.12 (4.03)	48.5 (4.43)^*^
	Control	47.52 (3.83)	47.44 (3.8)	47.53 (4.05)
Waist circumference (cm)	Diet	75.66 (4.31)	69.58 (3.02)^*^	74.23 (3.92)^*^
	Control	74.18 (3.54)	74 (4.5)	72.9 (4.53)^*^
Android fat (g)	Diet	937.92 (324.3)	249.56 (144.55)^*^	840.8 (306.8)
	Control	919.41 (327.73)	984.65 (379.41)	902.29 (350.3)
Resistance training (METh/wk)	Diet	45.31 (8.76)	46.1 (9.9)	42.25 (8.23)
	Control	33.61 (19.44)	28.59 (14.22)	32.11 (17.77)
Aerobic exercise (METh/wk)	Diet	13.95 (10.43)	22.3 (17.79)^*^	10.99 (12.19)
	Control	15.76 (23.81)	13.16 (14.54)	16.65 (25.61)
Energy intake (kCal/vrk)	Diet	2,348 (418)	1,664 (305)^*^	2,315 (549)
	Control	2,522 (528)	2,356 (432)	2,518 (380)
Energy intake (kCal/kg)	Diet	36.51 (6.54)	29.62 (5.49)^*^	37.8 (9.87)
	Control	39.6 (8.04)	36.76 (5.77)	39.74 (5.46)
Energy availability (kCal/kg/FFM/day)	Diet	36.3 (9.2)	24.6 (7.5)^*^	28.7 (8.8)^*^
	Control	40.5 (11.5)	38.6 (8.1)	44.8 (9.4)

*BMI, body mass index; FFM, fat free mass; kcal, kilocalories; METh/wk, metabolic equivalent hours per week. Values are presented as mean (standard deviation, SD) unless otherwise noted. Means and SDs are calculated for the study sample n = 42*.

* *Statistically significant difference from baseline (P < 0.05) within-group comparisons. P-values are calculated with generalized estimating equations and age was accounted for in the model. Dietary diaries during the weight regain period were collected from the middle of the **MID–POST** measurements*.

### Prolonged Energy Deprivation and Intense Exercise Results in Altered Blood Cell Distribution and Hematopoiesis Regulation

**Figure 2:**
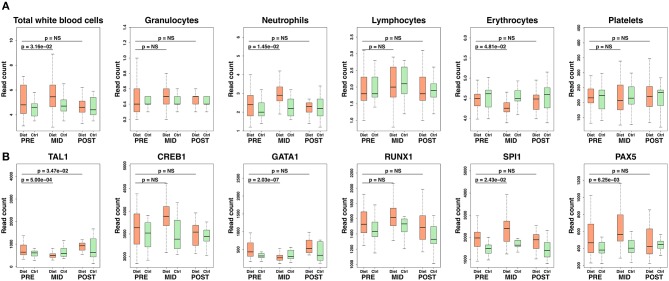
Transcription regulators of hematopoiesis and blood cell count alteration following intense weight loss. Here, we demonstrate alterations occurring in hematopoiesis regulation and subsequent changes in circulating blood cells. In particular, suppressed differentiation in erythroid lineage and induced activity of leukocyte progenitor cell lines (myeloid, lymphoid) were suggested by increased *SPI1* and reduced *GATA* transcription factor expression levels. Panel **(A)** depicts absolute levels of different categories of quantified blood cells, whereas panel **(B)** demonstrates downstream modulation of transcription factors affecting blood cell formation. Ctrl, control; NS, not significant (*P* > 0.05 on panel **(A)**, false discovery rate, FDR > 0.05 on panel **(B)**. Granulocytes depicted in the figure include basophils, eosinophils, and monocytes (i.e., mixed cells). Box-plot elements are defined as follows: center line, median; box limits, upper and lower quartiles; whiskers, 1.5× interquartile range.

As shown in Figure 2, weight loss resulted in a significant (*P* < 0.05) increase in absolute numbers for two immune cell categories in the diet group: neutrophils and total WBC count, where circulating neutrophil numbers accounted for most of the increase in WBCs (Figure 2; Supplementary Table 1). Also, a *relative* decrease in the percentage of lymphocytes (β = −5.10 ± 2.31, *P* = 0.03) was observed in the diet group, explained also mostly by the augmentation in neutrophil numbers (Supplementary Table 1). These changes in immune cell distribution were accompanied by a reduction in erythrocyte and platelet counts thus suggesting a wider array effect on blood cell proliferation and hematopoiesis (Figure 2; Supplementary Table 1). The prolonged strict diet and rigorous exercise had no long-term effects on blood cell distribution as the numbers in WBC categories, erythrocytes, and platelets recovered back to baseline during the weight regain period in the diet group (Figure 2; Supplementary Table 1). As expected, no significant changes (*P* > 0.05) were detected across time points in the control group (Supplementary Table 2).

**Figure 3:**
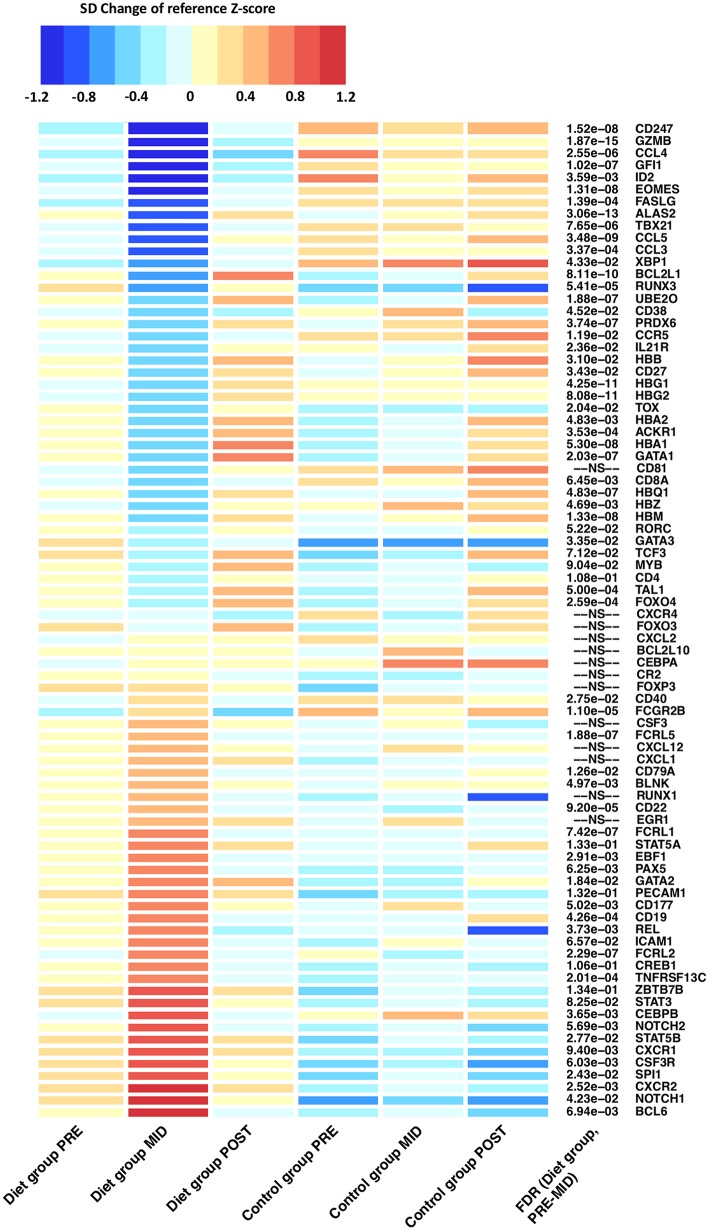
Differentially expressed genes related to innate and adaptive immune system function after the prolonged period of low-energy availability. Heat map of differentially expressed genes depicts alteration in RNA expression levels that belong to immune function related pathways after the weight loss period when compared with controls (**PRE–MID**). Heat map is derived from DESeq2 normalized expression levels (read counts) that are represented as standard deviation (SD) change from the reference Z-score. The baseline calculated Z-score values (**PRE**) from both the diet and control groups were pooled together and set as the reference level to which each individual group/time-point level was compared. On the heat map, blue colors indicate decrease and red colors increase in gene expression level compared with the calculated reference value. FDR = false discovery rate. Adjusted *P-*values are represented in front of each HGNC gene symbol name. FDR > 0.2 was set as not significant (NS).

The above findings on blood cell distribution were further supported by differential expression in individual key transcription factors including *TAL1, RUNX1, CREB1, SPI1*, and *GATA1* regulating hematopoietic stem cell (HSC) proliferation and survival (Figure 2; Supplementary Table 3) (20–23). In particular, suppressed differentiation in erythroid lineage and induced activity of myeloid and lymphoid progenitor cell lines were suggested by increased *SPI1* (PU.1) and reduced *GATA1* expression levels (Figure 2; Supplementary Table 3). Suppression of erythroid progenitor cell line proliferation and reduced erythrocyte and platelet numbers were further supported as downregulation of genes responsible for hematopoiesis regulation (e.g., *ACKR1*); hemi- and hemoglobin production (e.g., *ALAS2, HBG1, HBG2, HBA1, HBD, HBM, HBQ1, UBE2O*) were also detected (Figure 3; Supplementary Table 3) (12). Furthermore, closer examination of transcription factors suggested alteration in the development of various leukocyte producing HSC progenitor cell lines (e.g., *ERG1, C/EBP*α*/*β, *Gfi-1, GATA2, GSF3R, NOTCH1/2, PAX5, EBF*) (Figure 3) (24, 25).

Moreover, after intense exercise-induced weight loss, evidence of increased metabolic stress by reactive oxygen species (ROS) on the pool of HSCs was implied through downregulation of FOXO transcription factors (e.g., *FOXO3, FOXO4*) which are essential for protecting HSCs from ROS dependent HSC aging (Figure 3) (26, 27). Furthermore, augmented ROS induced apoptosis in HSCs was suggested by distinct downregulation anti-apoptotic transcription factors (e.g., *BCL2L1, BCL2L10, PRDX6*) (28, 29). No long-term effects were detected on HSC regulating transcription factors as expression levels reverted back to baseline during the weight regain period (Figure 3).

Together, these data on leukocyte derived transcriptomics and blood cells implied intense exercise and low-energy availability to augment leukocyte-skewed HSC proliferation and mobilization from bone marrow—subsequently resulting in increased total numbers of circulating leukocytes and reduced erythrocyte numbers. Data from leukocyte transcriptomics also suggested accumulated metabolic stress that could possibly contribute to the observed HSC proliferation, and in theory ROS dependent HSC aging.

### Inhibition of T-Lymphocyte Proliferation and Biased CD4 T-Helper Response May Participate in Mediating Immunodeficiency After a Prolonged Period of Low-Energy Availability

Next, we evaluated whether the effects of prolonged energy deprivation and intense exercise influenced lymphocyte maturation and activation—key regulators in priming adaptive immunity. As a consequence of the intense weight loss period, transcriptomic analysis revealed distinct downregulation (*q*-value < 0.05) of the adaptive immunity pathway. We further assessed this pathway by examining individual adaptive immunity associated genes that were differentially expressed (Figure 3).

Evidence of T-lymphocyte mediated immunosuppression induced by low-energy availability was suggested by suppressed markers of peripheral T-lymphocyte differentiation and activation. A multiplicity of key regulating genes (e.g., *GATA3, ZBTB7B, RUNX3, MYB, TOX, EOMES, GZMB, FASLG*) were differentially expressed after the intense weight loss period suggesting inhibition of the two major T-lymphocyte proliferation cell lines: helper (T_H_)/CD4 and cytotoxic/CD8 lymphocytes (Figure 3; Supplementary Table 3) (30). In particular, suppressed lineage fate of cytotoxic CD8 T-lymphocytes was implied by downregulation of *RUNX3* and upregulation of *ZBTB7B* (ThPok) expression levels (Figure 3). Suppression of cytotoxic CD8 T-lymphocyte lineage fate was further indicated by significant (*q*-value < 0.05) downregulation of the pathway involved in the Class I major histocompatibility complex (MHC) mediated antigen processing and presentation. Aforementioned pathway is principally involved in CD8 T-lymphocyte antigen presentation and subsequent maturation (30). In addition, an antigen pathway associated with ubiquitination and proteasome degradation was also downregulated (*q*-value < 0.05) in a similar fashion, further supporting the notion that prolonged energy deprivation and intense exercise alter gene expression of genes related to antigen presentation, proliferation, and activation of immune cells.

T-lymphocyte mediated immunosuppression was further characterized by modulation and skewing of CD4 T_H_ subset proliferation that was suggested by the differential expression of “master” transcription factors *TBX21* and *GATA3* that regulate the generation of specific CD4 T_H_ subsets, particularly T_H_1 and T_H_2 cells (Figure 3). These findings imply suppression of the CD4 T_H_1 cell line proliferation, concomitant with the predominant T_H_2 response (31). Suggested predominant T_H_2 response was further supported by increased levels of eotaxin (β = 28.95 ± 5.69, false discovery rate (FDR) = 1.36 × 10^−5^), chemokine related to eosinophils, which are leukocytes that have repeatedly been associated with the effector arm of T_H_2 immune responses (32). Suppressed T_H_1 proliferation was further implied by downregulation of the aforementioned transcription factors (e.g., *EOMES, RUNX3, STAT4*) (Figure 3). Lastly, concomitant with the suggested augmentation in T_H_2 response, induced differentiation of the T-lymphocyte follicular helper (T_FH_) cell line was suggested through upregulation of master regulator *BCL6* (Figure 3) (33). T_FH_ cells are an effector group specialized in B-lymphocyte help and germinal center development.

Together, these results from transcriptional regulation and eotaxin levels are assumed to indicate attenuated T-lymphocyte maturation, an increase in the ratio of CD4/CD8 T-lymphocytes, and biased CD4 T_H_2 helper cell response after the prolonged period of low-energy availability and intense exercise.

### Prolonged Exposure to Low-Energy Availability May Predispose to Immunosuppression Through Inhibition of B-Lymphocyte Maturation and Proliferation

Adaptive immunity was evaluated further as we next examined whether suggested suppression of T-lymphocyte maturation also extended to B-lymphocyte populations. Similarly to T-lymphocytes, suppressed markers of B-lymphocyte proliferation was indicated as a wide array of genes (e.g., *CD22, FCGR2B, FCRL2, FCRL5*) known to participate in inhibitory B-cell receptor (BCR) signaling, mediator of B-lymphocyte proliferation, were upregulated (Figure 3; Supplementary Table 3). To this end, upregulation (*q*-value < 0.05) of the BCR signaling pathway was detected—an important regulator of immature B-lymphocyte proliferation in both bone marrow and periphery (Figures 2, 3) (34). However, findings on induced transitional B-lymphocyte production were also implied by upregulation of genes coding for *CD19* and *CD79a*, proteins widely expressed during all phases of B-lymphocyte development on the cell surface, until terminal differentiation into plasma cells (Figure 3; Supplementary Table 3).

Furthermore, differential expression of transcription factors suggested suppressed B-lymphocyte maturation and plasma cell production (Figure 3) (34, 35). Reduced maturation of plasmablasts and plasma cells was implied through upregulation of *BCL-6* and downregulation of *BLIMP* and *XBP1*—transcription factors regulating B-lymphocyte maturation to immunoglobulin producing plasma cells (Figure 3; Supplementary Table 3). In accordance with these findings, we detected reduced expression in *CD27* and *CD38* genes responsible for coding surface proteins, mainly observed on the surface of mature plasmablasts and plasma cells (Figure 3; Supplementary Table 3). Lastly, augmented expression of *NOTCH-1/2* and consequent downregulation of *E2A*, known inductors of marginal zone B-lymphocyte development, suggested a possible expansion in the non-circulating B-lymphocyte pool (Figure 2). This was further implied by differential expression of *BCL6, CD40, FCRLA*, and *BAFF-R*, which are known to promote expansion of a memory B-lymphocyte pool and germinal center B-lymphocyte development rather than plasma cell maturation (33).

Together, these omics data suggested reduced B-lymphocyte maturation to antibody secreting plasma cells and promoted germinal center development and possible expansion of the non-circulating B-lymphocyte pool as a consequence of the intense weight loss achieved by prolonged low-energy availability and intense exercise.

### Altered Regulation of B-Lymphocyte Proliferation Is Accompanied by Reduced IgG Antibody Levels and Modulated IgG Glycosylation After the Prolonged Exposure to Low-Energy Availability

Since mature plasma cells are responsible for producing and secreting the most common antibody, IgG, in the human circulation, we next determined *isolated* IgG levels and IgG N-glycan composition to assess further changes in: (i) IgG antibody mediated immune system function and (ii) anti- and pro-inflammatory activity of IgG N-glycan peaks that could affect also the binding affinity of IgG to BCRs. Due to prolonged energy deprivation and intense exercise in the diet group, we observed a significant reduction in the *total isolated* IgG levels (β = −0.04 ± 0.01, FDR = 4.85 × 10^−3^), supporting the above evidence of inhibited B-lymphocyte proliferation to mature plasma cells.

**Figure 4:**
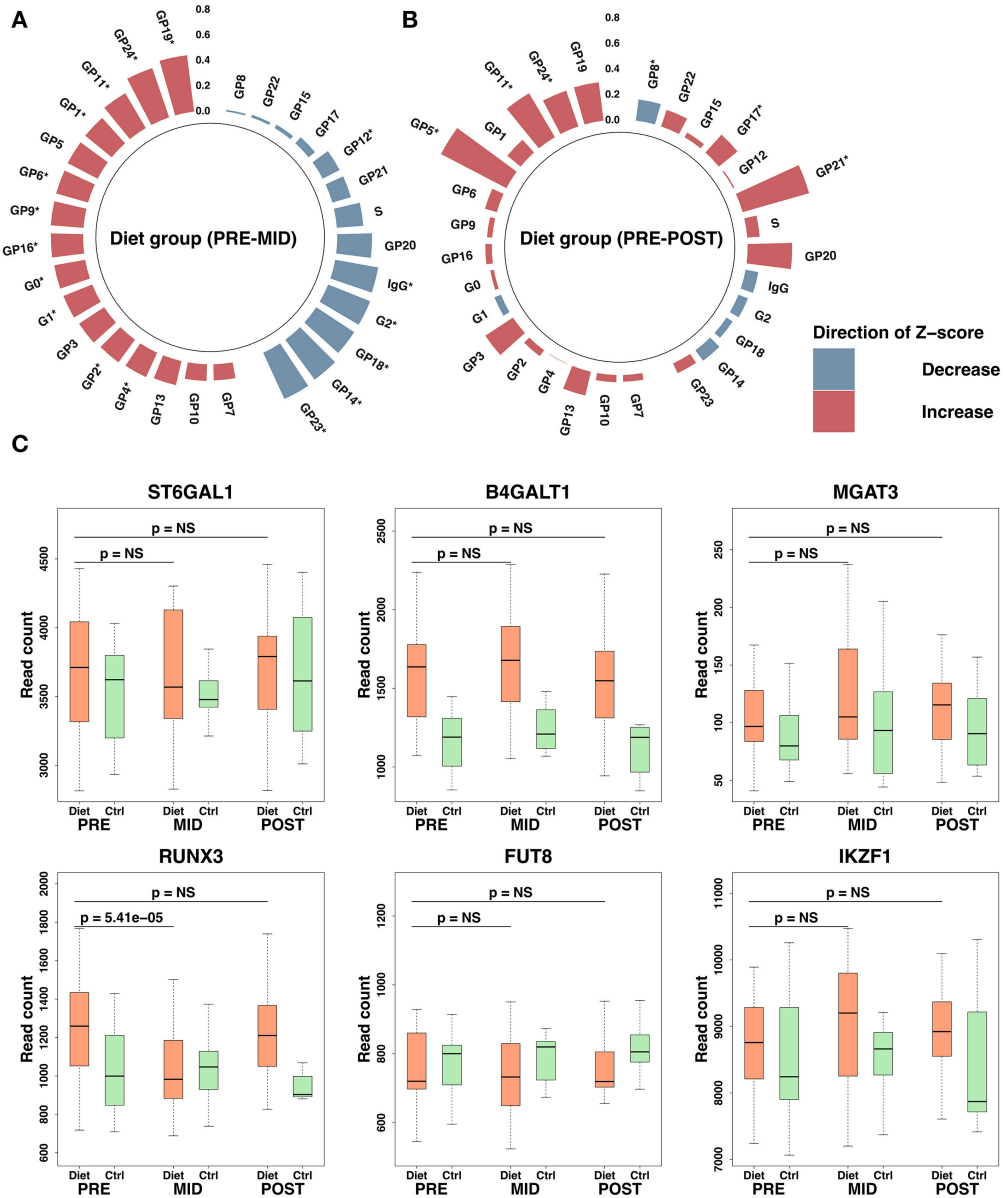
Immunoglobulin G (IgG) glycosylation peak modulation and expression changes in associated transcriptomic regulators after the intense weight loss period. Overall, this figure shows IgG glycosylation changes in the diet group during the study. Polar plots are derived from IgG glycosylation peak raw values where outliers based on four standard deviations (SDs) from the mean have been excluded. Plotted values are represented as SD change from the set reference *Z*-score. Red color indicates increase and blue a decrease compared with the reference *Z*-score. Height of the bars depicts the *Z*-score level, and the scale is plotted on the figure vertically. Significant time-dependent changes are assigned on the figure with an asterisk (^*^). IgG glycosylation peaks are ordered based on panel **(A)** for the diet group. *Z*-score reference values for the diet group were determined from the baseline values. Panels **(A,B)** demonstrate diet group IgG glycosylation changes after the weight loss period (panel **A**, **PRE–MID**) and after the whole study period (panel **B**, **PRE–POST**). Panel **(C)** demonstrates expression levels observed in transcription factors associated with IgG glycosylation. *P-*values in panel **(C)** have been adjusted for multiple testing by using false discovery rate (FDR). Significance threshold was set to FDR < 0.05. Box-plot elements are defined as follows: center line, median; box limits, upper, and lower quartiles; whiskers, 1.5 × interquartile range.

Changes in the abundance of distinct glycan peaks were mostly observed after the weight loss period (FDR < 0.05) (**PRE–MID**), returning mostly to the initial baseline values following the weight regain period (**MID–POST**) (Figure 4; Supplementary Table 4). Glycan peaks containing digalactosylated (GP12 and GP14) and sialylated N-glycans without bisecting N-acetylglucosamine, i.e., GlcNAc (GP17, GP18, GP21, and GP23) as well as derived traits G2 (GP12–GP15) and S (GP16–GP24 except GP20), were significantly decreased (FDR < 0.05) after the weight loss period (Figure 4; Supplementary Table 4). In contrast, agalactosylated (GP1, GP2, GP4, and derived trait G0) and monogalactosylated (GP8 and GP9) glycans, glycans with bisecting GlcNAc (GP6, GP11, GP19, and GP24), FA2G1S1 (GP16), and M5 (GP5) significantly increased (FDR < 0.05) in the diet group after the weight loss period (Supplementary Table 4).

No significant (FDR > 0.05) time-dependent changes were observed either in IgG glycome composition (Supplementary Table 5) or total isolated IgG levels (β = −0.01 ± 0.02, FDR = 0.85) (**PRE–POST**) in the control group during the study period. Detected changes in IgG glycosylation after the weight loss were supported by evidence of differential expression in genes coding transcription factors and in glyco- and galactosyltransferases (e.g., *ST6GAL1, B4GALT1, MGAT3, RUNX3, FUT8, IKZ1*) known to participate in the modulation of IgG glycosylation (Figure 4) (36). In addition, more distinct impact was detected in other galactosyltransferases (e.g., *ST6GALNAC3/4/6, B4GALT4/5/6, B3GNT5/8, GALNT5, GALNTL6*) that may also play a role in IgG glycosylation (Supplementary Table 3).

Together, these data showed that suggested alteration in B-lymphocyte proliferation subsequently was accompanied by reduced IgG levels, overall IgG glycosylation status alteration toward pro-inflammatory activity (galactosylation ↓, bisecting GlcNAc ↑), and reduced IgG affinity (sialylation ↓) with specific BCRs.

### Prolonged Energy Deprivation and Intense Exercise Suppresses Signaling and Regulation of IgE Antibody Dependent Immune Reactions

The above alterations in IgG mediated immunity were accompanied by a significant (*q*-value < 0.05) downregulation of the Fc epsilon receptor (FceRI) signaling pathway—the basophil, eosinophil, and mast cell dependent immunoglobulin E (IgE) antigen-specific signaling pathway that mediates the release of potent inflammatory substances and consequent allergic inflammation (37). Together, these results of downregulated IgE and IgG antigen-specific signaling pathways enforce the perception of suppressed antibody mediated immunity.

### A Prolonged Period of Low-Energy Availability Suppresses the Release of Inflammation Markers and Both the Innate and Adaptive Immune System Associated Chemokine Production

**Figure 5:**
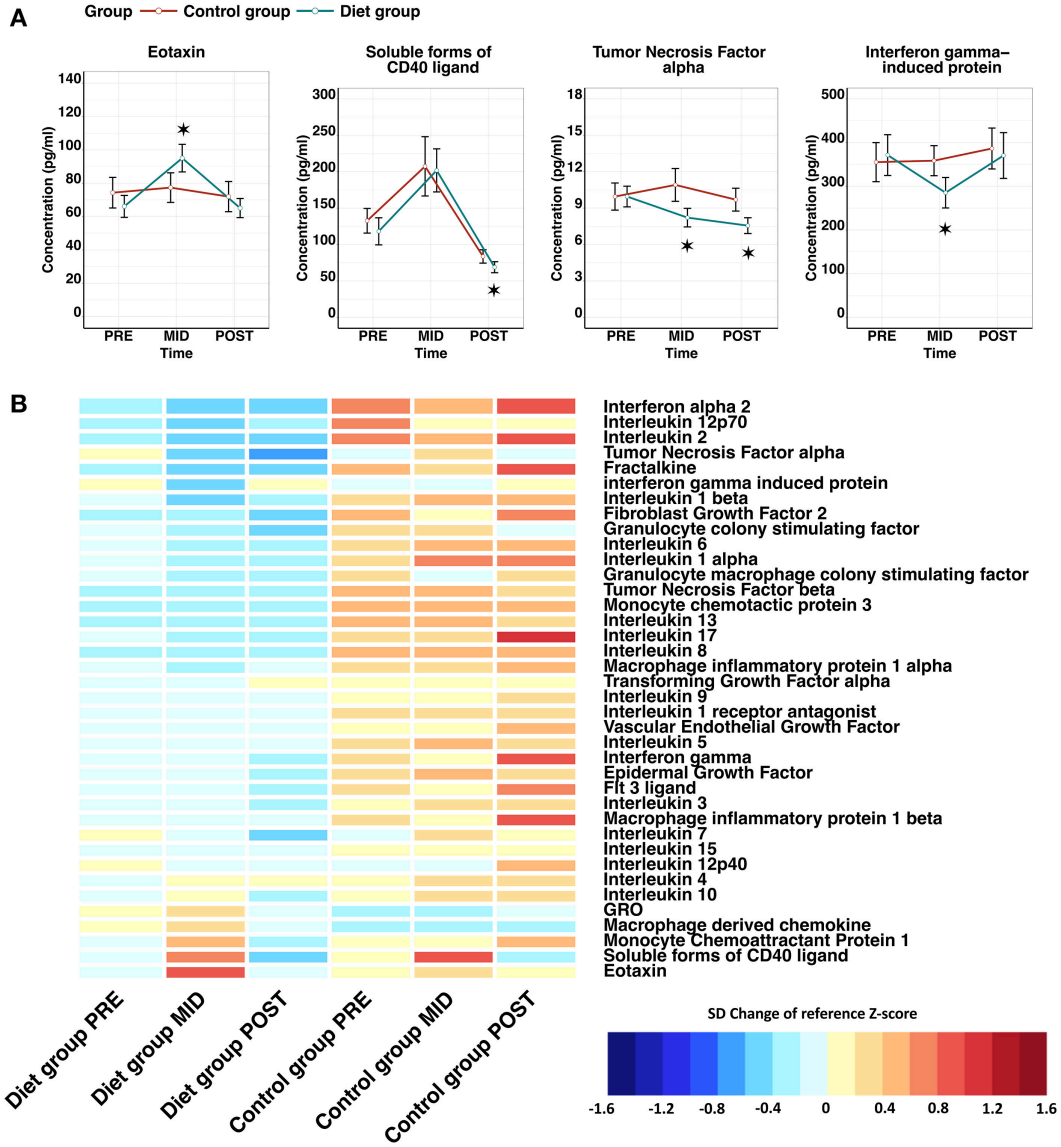
Effect of low-energy availability on cytokine profile alteration in the study participants. Panel **(A)** line plots show that cytokines were altered significantly in a time-dependent manner during the weight loss period (**PRE–MID**) in the diet group. The red line represents the control group, whereas the blue line depicts the diet group. Error bars are depicted as standard error (SE). Significant time-dependent changes compared with baseline (**PRE**) are depicted with asterisks, where ^*^ = false discovery rate (FDR) < 0.05. Panel **(B)** depicts a heat map of the entire measured cytokine profile, including the ones with significant time-dependent changes shown in the panel **(A)**. Cytokine levels are represented as standard deviation (SD) change from the reference *Z*-score. Baseline calculated *Z*-score values (**PRE**) from both the diet and control groups were pooled together and set as the reference level to which each individual group/time-point level was compared. On the heat map, blue colors indicate decrease and red colors increase in cytokine level compared with the calculated reference value. FDR adjusted *P-*values for within-group time-point comparisons are depicted in front of the cytokine names. Panel **(B)** demonstrates clearly that despite only a handful of significant time-dependent changes seen in panel **(A)** in the diet group, the overall cytokine levels are notably lower in the diet group when compared with the control group.

Suppression of innate immunity was also suggested by downregulation (*q*-value < 0.05) of the innate immunity pathway in the transcriptomic analysis. To further assess adaptive and innate immune cell interactions and relationships, cytokine analysis was performed, where we observed most clearly an indication of (i) reduced pro-inflammatory chemokine secretion and (ii) induced myeloid cell targeted chemotaxis. Specifically, a significant reduction in the levels of tumor necrosis factor alpha (TNF-α) (β = −1.74 ± 0.53, FDR = 1.26 × 10^−2^) and interferon-gamma induced protein 10 (IP10) (β = −86.10 ± 28.96, FDR = 2.80 × 10^−2^) was detected after the weight reduction period (**PRE–MID**) in the diet group (Figure 5; Supplementary Table 6). Furthermore, evidence of higher circulating levels of monocyte chemoattractant protein 1, macrophage derived chemokine, and GRO were detected, important chemokines mediating myeloid cell line proliferation and chemotaxis (Figure 5).

As with other measured omics, the majority of cytokines showed a similar tendency to revert back to baseline levels during the weight regain period (**MID–POST**) (Figure 3). The only exception was TNF-α (β = −2.40 ± 0.54, FDR = 3.28 × 10^−4^), the levels of which decreased significantly, reaching even lower levels than at baseline (**PRE–POST**) and thus supporting the possibility of potential prolonged benefit (Figure 5). In the control group we did not observe any significant time-dependent changes apart from in soluble CD40 ligand (sCD40L) (β = 88.69 ± 22.27, FDR = 3.26 × 10^−3^), which depicted a similar pattern as the diet group when examining the whole study period (Figure 3; Supplementary Table 7). Along with chemokine profile changes, we also observed the differential expression of several genes (FDR < 0.05) regulating corresponding chemokine receptor (e.g., *CXCR1, CXCR2*) production, further suggesting alteration in chemokine and chemokine receptor interaction in innate immunity (Table 2).

**Table 2:** Expression level changes of chemokine receptor genes after weight loss in the diet group.

**HGNC symbol**	**Ligand of corresponding receptor**	**Base mean**	**log2FC**	**log2FC SE**	**Stat**	***P* value**	**FDR**
IL2RB	IL-2	1325.73	−0.48	0.08	−5.72	1.05*E*−08	1.08*E*−06
IL12RB2	Interleukin 12	123.32	−0.39	0.08	−4.73	2.30*E*−06	1.16*E*−04
IL12RB1	Interleukin 12	504.36	−0.20	0.06	−3.60	3.18*E*−04	5.78*E*−03
CCR5	MIP 1 alpha and beta	433.35	−0.19	0.06	−3.33	8.68*E*−04	1.19*E*−02
IL21R	Interleukin 21	781.75	−0.13	0.04	−3.05	2.27*E*−03	2.36*E*−02
IL10RA	Interleukin 10	6853.18	−0.11	0.03	−3.26	1.11*E*−03	1.41*E*−02
CCR2	Monocyte chemotactic protein 3, MCP-1	2667.91	0.14	0.05	2.98	2.86*E*−03	2.77*E*−02
IFNAR2	Interferon alpha/beta	2588.25	0.16	0.04	4.02	5.75*E*−05	1.52*E*−03
CD40	sCD40L	211.27	0.17	0.06	2.99	2.83*E*−03	2.75*E*−02
IL10RB	Interleukin 10	1807.81	0.18	0.06	3.17	1.53*E*−03	1.79*E*−02
CSF2RA	GM-CSF	1212.22	0.19	0.07	2.80	5.08*E*−03	4.13*E*−02
IFNGR2	Interferon gamma	3209.86	0.20	0.07	2.91	3.66*E*−03	3.29*E*−02
IL6R	Interleukin 6	8531.50	0.22	0.06	3.51	4.50*E*−04	7.43*E*−03
IL6R	Interleukin 6	8531.50	0.22	0.06	3.51	4.50*E*−04	7.43*E*−03
IFNAR1	Interferon alpha/beta	3012.60	0.23	0.06	4.06	4.96*E*−05	1.38*E*−03
TNFRSF1A	Tumor necrosis factor alpha	3839.61	0.24	0.08	3.10	1.91*E*−03	2.09*E*−02
LTBR	Tumor necrosis factor beta	732.44	0.26	0.08	3.25	1.17*E*−03	1.47*E*−02
IL1R2	Interleukin 1	1439.90	0.28	0.10	2.78	5.45*E*−03	4.33*E*−02
FLT3	Fit 3 ligand	210.37	0.29	0.09	3.04	2.37*E*−03	2.43*E*−02
CSF3R	G-CSF	25828.21	0.29	0.08	3.58	3.40*E*−04	6.03*E*−03
IL13RA1	Interleukin 13 and 4	3779.96	0.31	0.07	4.69	2.73*E*−06	1.34*E*−04
CCR1	MIP 1 alpha	1646.34	0.31	0.09	3.54	4.04*E*−04	6.82*E*−03
CXCR1	Interleukin 8	8567.92	0.36	0.11	3.42	6.27*E*−04	9.40*E*−03
IL1RAP	Interleukin 1	2460.93	0.39	0.08	4.71	2.48*E*−06	1.22*E*−04
CXCR2	Interleukin 8, MIP-alpha, MIP-beta, GRO	14397.73	0.41	0.11	3.87	1.09*E*−04	2.52*E*−03
IL1R1	Interleukin 1	357.11	0.49	0.11	4.61	4.11*E*−06	1.92*E*−04

*log2FC, log2FoldChange; log2FC SE, log2FoldChange standard error; Stat, Wald statistic; FDR, false discovery rate; MIP, macrophage inflammatory protein; MCP-1, monocyte chemoattractant protein 1; GM-CSF, granulocyte-macrophage colony-stimulating factor; G-CSF, granulocyte-colony stimulating factor. Only chemokine receptor genes with significant expression changes as a consequence of severe weight loss are included in the table. Results were derived from statistical analysis using Wald-test*.

Together, these omics findings imply also downregulated innate immunity responses by suppressed release of inflammation markers and overall chemokine production after the prolonged period of low-energy availability and intense exercise. In the end, we note that the majority of the observed changes in the markers of both adaptive and innate immunity returned to baseline levels during the recovery period (**MID–POST**).

## Discussion

This integrated high-throughput omics study showed that vigorous physical training together with reduced energy intake leading to intense weight loss in females of previous normal weight had a substantial effect on the function of both innate and adaptive immunity. Our new discoveries consisted of molecular pathways associated with suppressed immune cell proliferation and loss of immune cell function by reduced antibodies, and chemokine secretion that may explain altered immunity and immunosuppression following prolonged intense exercise and low-energy availability leading to reduced adiposity.

It is widely accepted that obesity, adipose tissue dysfunction, and weight gain have been associated with elevated levels of the *total* number of leukocytes and especially neutrophils, which are markers for systemic low-grade inflammation and a risk factor for coronary heart disease (38–40). On the other hand, exercise training and weight loss have been shown to have an opposite effect in overweight individuals (41–45). Similarly, even further weight loss below normal levels of body weight (e.g., in anorexia nervosa) has reduced the leukocyte concentration (46). This is in contrast to our observation of augmented neutrophil numbers after the diet (Figure 2). An attenuated level of systemic inflammation was indicated by cytokines (e.g., TNF-α ↓, IP10 ↓, Figure 5) and previously published findings on metabolomic markers [high-density lipoprotein cholesterol ↑, α_1_-acid glycoprotein ↓, high-sensitivity C-reactive protein (S-hs-CRP) levels ↓ (19)] after the weight loss thus alleviating doubts of infection, inflammation, or disease induced leukocytosis. Altogether, discrepancies between previous findings and our results suggest that both starting weight and whether intensive weight loss is attained through dietary restriction only or is combined with intense exercise contribute notably to the circulating number of leukocytes.

In accordance with the augmented total number of leukocytes, our integrated omics findings suggest leukocyte-skewed augmentation in HSC proliferation from bone marrow following the intense weight loss period (Figure 2; Supplementary Table 1). Dietary restriction has been characterized as an important promoter of HSC quiescence, whereas, in contrast, physical exercise has been shown to be one of the most potent stimuli for the proliferation of HSCs from bone marrow to the circulation (47–49). It can be speculated that loss of quiescence and excessive proliferation of HSC through a very high amount of exercise with energy restriction may accelerate the exhaustion of HSC niches thus in theory promoting HSC aging (50–53). Subsequently, in accordance with our findings (Figures 2, 3), aging phenotypes of HSCs have been characterized by an increase in the pool size of HSCs, skewing toward myeloid-biased HSCs vs. lymphoid-biased HSCs, enhanced mobilization from the bone marrow into the circulation, and suppressed erythroid lineage proliferation (52). Furthermore, enhanced HSC proliferation and turnover from bone marrow suggested by us were also corroborated by loss of overall bone mass after intense weight loss (12). Reduction in bone mineral density (e.g., osteopenia) together with low-energy availability and amenorrhea/oligomenorrhoea are part of female athlete triad—a syndrome seen in females participating in sports that emphasize leanness or low body weight, such as physique sports. As reported previously by Hulmi et al., the diet group participant had reduced levels of sex hormones together with reported changes in their menstrual function during the weight loss period (12). Based on previous studies and our findings, we argue that the combined prolonged stress of high-intensity training and low-energy availability without sufficient recovery leading to increased metabolic stress augments neutrophil numbers and may have the potential to promote HSC proliferation and partially similar phenotype characteristics as observed in HSC aging.

Malnutrition, nutritional deprivation, and low-energy availability are thought to be one of the major causes of immunodeficiency where the most common immune defects are atrophy of lymphoid tissues (e.g., thymus), reduced maturation of T-lymphocytes, an imbalance in the ratio of CD4/CD8 T-lymphocytes, and predominant T_H_2 helper cell response (54–59), alterations that were consequently also suggested by the leukocyte transcriptome in our study (Figures 3, 5). These immune system defects have been previously reported to be mediated by reduced levels of leptin caused by low-energy availability and exhaustive exercise (12, 60)—a response also observed in the diet group participants of our study after the weight loss period (**PRE-MID**). On the other hand, leptin administration and moderate levels of exercise have successfully prevented these immune system alterations and consequent immunosuppression (55). In accordance with these observations from previous studies, reduced levels of exercise and increased energy intake leading to weight regain (**MID-POST**) lead to the elevation in leptin levels in the diet group participants ^12^. Reversion in leptin levels were accompanied by normalization in the expression of regulatory genes associated with T-lymphocyte proliferation and T_H_ response in our study (Figures 3, 5). Altogether, these findings suggest that dietary restriction and intensive exercise may cause suppressed peripheral T-lymphocyte proliferation and predominant T_H_2 helper cell response leading to immunodeficiency through reduction in leptin levels (55).

Previous studies suggest that low-energy availability negatively impacts also B-lymphocyte maturation; however, the underlying mechanisms have not been elucidated in detail. It has been implied that both starvation and HSC aging arrest B-lymphocyte development, diminish the pool of naive B-lymphocytes, and expand the germinal center cell pool of memory B-lymphocytes (61). Similar responses to low-energy availability were suggested by the integrated omics used in our study, as expression patterns of leukocyte transcriptome implied suppressed B-lymphocyte proliferation, skewed-germinal center expansion, diminished production of mature IgG producing plasma cells, and reduced IgG antibody production (Figures 3, 4). To further corroborate our findings, a similar loss of function and diminished antibody production have been observed in aging immune systems and in highly trained athletes during prolonged periods of intensive exercise training without sufficient recovery (62). Suppression of B-lymphocyte proliferation and secretion function have been proposed to be mediated by the previously discussed alteration in leptin levels (59, 63). In the end, we argue that reduction in leptin levels may at least in part also cause these modulations in B-lymphocyte maturation and subsequent antibody secretion. Therefore, our findings reinforce the perception that the impact of dietary restriction and intense exercise may extend also to B-lymphocyte population proliferation and function thus contributing to mechanisms behind immunosuppression following intense weight loss in previously normal weight individuals.

Lean phenotype, voluntary weight loss, anti-inflammatory skewed cytokine profile, together with enhanced CD4 T_H_2 response, as suggested by our integrated omics findings, have been shown to be associated with innate immune cell modulation leading to a regulatory/wound-healing macrophage (M2/M3) dominant profile (64–67). Regulatory/wound-healing macrophages have the ability to dampen immune response and limit inflammation (67) thus further reinforcing our observation of alleviated systemic inflammation and downregulated immunometabolism. However, although classically activated macrophages (M1) and consequent pro-inflammatory cytokine flux have been associated with adverse health effects such as chronic diseases, obesity, and disease related weight loss, they also mediate positive effects on the eradication of premalignant tumor cells (67–69). Thus, these observations underline the delicate existing homeostasis that needs to be sustained between different aspects of immune system activation and suppression to prevent adverse health effects. Despite the beneficial direction of changes observed in the systemic inflammation and immune responses of the adaptive and innate immune system in our study, it remains to be elucidated whether these observed alterations mediate additional health benefits or whether they lead to adverse disruption of immune system homeostasis by excessive immune system downregulation.

Severe dietary restriction and malnutrition in individuals with low body weight (e.g., anorexia nervosa) have been previously associated with increased risk of various autoimmune diseases (70). In addition, mucosal abnormalities/disturbances such as thinned mucosa, infiltration of immune cells, and increased intestinal permeability have been detected (71). Consequently, we observed increased pro-inflammatory activity of IgG (galactosylation ↓, bisecting GlcNAc↑), reduced affinity of IgGs (sialylation ↓) with specific BCRs (e.g., Fc receptor-like proteins, FCRLs), altered IgE mediated signaling, higher levels of eotaxin, and suggested predominant T_H_2 response (Figures 3–5). These responses have been associated with immune system dysregulation and autoimmune diseases related to the lungs and intestines, such as asthma, inflammatory bowel disease, and gastrointestinal allergic hypersensitivity (72, 73). Molecular pathways associated with mucosal immunity could to some extent explain the mechanism behind higher risk of infection during periods of intense exercise with low-energy availability (60, 74–76). Altogether, multiple aspects of suppressed immune function together with findings related to autoimmunity implied by our integrative omics data provoke the question as to whether these immune system related alterations predispose to greater risk of autoimmune dysregulation and adverse immune system associated health outcomes in the long run (77).

Consistent with our findings, previous studies investigating gene expression pathways associated with adiposity and weight loss have revealed significant associations with genes related to immune system function (5, 78, 79). In addition to intense exercise, obesity is also associated with immune system dysfunction including impaired cell-mediated response and increased levels of systemic inflammation (1, 3, 80). Activation and upregulation of immune response pathways are evident in obese individuals when compared with lean individuals (78). Similarly, we showed that weight loss resulted in downregulation, and weight regain in upregulation, major immune function related pathways and associated genes (Figure 3). Altogether, current evidence suggests that energy availability and adiposity level tightly regulate RNA expression levels of immune function related pathways, which could partly explain immune system dysfunction in both obese individuals and vigorously exercising athletes with low quantities of fat mass and low-energy availability.

Comprehensive integrative system biological data sets and a longitudinal study design including both weight loss and weight regain periods and a control group were strengths of our study. Integrative immune function targeted omics in our study corroborated each other on a multitude of levels as findings on HSC regulation, blood cell numbers, leukocyte proliferation, and cytokine profile were convergent, and they therefore advance understanding of these underlying mechanisms mediating immunosuppression. Limitations of our study were that (i) RNA expression levels derived from leukocytes do not necessarily reflect actual levels of biologically active proteins in specific leukocytes and the function of the immune system; (ii) despite a longitudinal design and thus rather good statistical power, the sample size was relatively small; and (iii) we did not have available reported subjective data on incidence of infections or infection symptoms from the participants to further evaluate immunosuppression. Although, leukocyte derived RNA expression levels have been reported to dynamically reflect system wide biology (81)—more accurate flow cytometry analysis of WBCs are warranted in future studies to validate the changes indicated by our study in WBC populations. We also recognize the lack of measured resting energy expenditure as a possible limitation as differences at baseline metabolic rate may contribute to the immunometabolism responses of the diet group participants when subjected to prolonged intense exercise and low energy availability.

In conclusion, based on our integrated omics findings, we show that prolonged periods of low-energy availability and a high amount of exercise leading to weight loss have a significant effect on multiple levels of the immune system, while most of these changes can be reverted through a sufficient recovery period leading to weight regain. More studies are warranted to examine the effects long-term intensive exercise with low-calorie diet and repeated weight loss bouts on the function and molecular mechanisms of the immune system function. Our findings highlight and reinforce the perception that starting weight and the way in which weight loss is achieved (voluntary vs. involuntary, dietary restriction vs. exercise mediated) have a considerable effect on immune system modulation and health.

## Materials and Methods

### Study Participants and Design

We recruited young previously normal-weight female physique athletes to participate in the present weight loss study. A detailed description of study design, study participants, recruitment, and phenotyping methods was reported previously (12). Briefly, female physique athletes aim to achieve a highly-refined aesthetic appearance with high muscle definition and symmetry by drastically reducing body fat levels during a vigorous 2- to 5-month progressive competition diet routine, followed by a weight regain period. Exclusion criteria for the study were: (i) prevalent diagnosed chronic disease, (ii) prescribed medication (e.g., thyroxine) excluding contraception, and (iii) individuals with <2 years of resistance training experience. In the end, study sample size and power was determined by considering (i) the evidence from previous longitudinal studies (82) and (ii) the number of individuals that would be feasible to measure and analyze throughout the study protocol.

This weight loss study initially included a total of 60 young previously normal-weight female amateur physique athletes of Caucasian origin competing or aiming to compete in national “Bikini” or “Body fitness” sports who were matched on weight, age, height, and reported training experience (Figure 1). Study participants volunteered into the control group (*n* = 30) or the diet group (*n* = 30), where athletes had an average of ~3 years of training experience. Individuals volunteered into the diet group had on average competed once (from 0 to 4 competitions) before this study regimen. To study the impact of intense exercise and low-energy availability leading to fat mass loss, we examined these individuals (age 27.5 ± 4.0 years, BMI 23.4 ± 1.7 kg/m^2^) at three test time points: (i) baseline tests were conducted before the weight loss regimen began (**PRE**), (ii) immediately after the weight loss period 21.1 ± 3.1 weeks (**MID**), and iii) after a weight regain period that averaged 18.4 ± 2.9 weeks in length (**POST**). In contrast to the diet group, the control group was instructed to maintain their typical weight and fitness lifestyle throughout the whole study period. At each experimental time point, participants of both groups went through a series of anthropometric and clinical tests. Serum and plasma samples were collected from the physique study population at all three time-point measurements at the same time of day after at least 8 h of fasting. The Ethical Committee at the University of Jyväskylä approved the study protocol and all participants gave written informed consent in accordance with the Declaration of Helsinki.

Of the 60 study participants who started the study, a total of 10 athletes failed to complete the study regimen in a required manner (Figure 1). One control did not arrive for baseline testing (**PRE**) and the remaining nine participants (three from the diet group and six controls) were excluded for failing to follow the study instructions or because of a short duration of the weight regain period compared with the other participants. In addition, participants who lacked complete dietary records (*n* = 8) were excluded from the current omics study. Due to the high cost of large-scale data-set quantification, we only included samples in the analysis from individuals with minimal missing information (i.e., the quality of nutritional intake records). In total, we included samples from 42 participants (diet group *n* = 25, control group *n* = 17) in the bioinformatic analysis after the relevant exclusions.

### Anthropometric Measurements

Body composition and anthropometrics were assessed with dual-energy X-ray absorptiometry (DEXA, Lunar Prodigy Advance, GE Medical Systems, Lunar, Madison, WI, USA) (12). Waist circumference measurements were made from the midpoint of the lowest rib and iliac crest when breathing out. Waist circumference and waist:hip ratio were used to assess the quantity of visceral region fat in addition to the visceral fat mass measured by DEXA.

### Nutrient Intake

The physique study participants reported nutrient intake repeatedly using self-reported dietary diaries from representative days throughout the study: at baseline (**PRE**), after the weight loss period (**MID)**, and after the weight regain period (**POST**) (49). The diet group participants followed strict dietary routines during the weight loss period (**PRE-MID**). Thus, ~50% of these diet group participants reported all of their meals as their eating was very controlled. The remaining individuals reported fewer number of representative days throughout the weight loss period due to more flexible dietary routines. Values reported from **MID** timepoint represent the lowest energy intake achieved during the weight loss period by the diet group participants. The control group reported dietary diaries over 3 weekdays and 1 weekend day at the baseline (**PRE**), in the middle of the study (**MID**), and during the last part of the study (**POST**). From the latter period (**POST**) similar dietary diaries were also obtained from the diet group participants at the end of the study period (**POST**). Nutritional supplements were included in the dietary analysis. The food diaries provided by the participants were analyzed by dietary analysis software (Aivodiet, Flow-team Oy, Oulu, Finland).

### Physical Activity

The duration and intensity of daily physical activity were also reported by the physique study participants throughout the study (**PRE, MID, POST)** from which overall physical activity (METh/wk) was calculated.

### Venous Blood Sampling and Analysis

All the participants were asked to sleep for at least 8 h during the preceding night and were required to refrain from strenuous physical activity for at least 24 h before venous blood sampling at baseline (**PRE**), after the weight loss period (**MID**), and after the weight regain period (**POST**). Venous blood samples were taken from the antecubital vein and stored in serum tubes (Venosafe; Terumo Medical Co., Leuven, Hanau, Belgium) using standardized laboratory procedures. Fasting blood samples were taken from both the diet and control group after 8 h of fasting at each time point.

### White Blood Cell Differential Count Analysis

Whole blood samples were analyzed within 30 min. Total and differential WBCs were measured with Sysmex KX-21N (TOA Medical Electronics Co. Ltd, Kobe, Japan). Of the WBCs, neutrophils, lymphocytes, and mixed cells (monocytes, eosinophils, basophils, and immature precursor cells) were analyzed.

### Glycome and Cytokines

#### IgG Isolation, and Glycan Release and Labeling

The whole procedure was performed as previously reported by Pučić et al. (83). Briefly, IgGs were isolated from the plasma samples using a Protein G 96-well plate (BIA Separations, Slovenia). The isolated IgGs were denatured with the addition of SDS (Invitrogen, USA) and by incubation at 65°C. The excess of SDS was neutralized with Igepal-CA630 (Sigma-Aldrich, USA) and N-glycans were released following the addition of PNGase F (Promega, USA) in phosphate-buffered saline. The released N-glycans were labeled with 2-AB. The free label and reducing agent were removed from the samples using hydrophilic interaction liquid chromatography solid-phase extraction (HILIC-SPE). Glycans were eluted with ultrapure water and stored at −20°C until use.

#### Ultra-Performance Liquid Chromatography

Fluorescently labeled N-glycans were separated by HILIC on an Acquity UPLC instrument (Waters, USA) consisting of a quaternary solvent manager, sample manager, and an FLR fluorescence detector set with excitation and emission wavelengths of 250 and 428 nm, respectively. The instrument was under the control of Empower 3 software, build 3471 (Waters). Labeled N-glycans were separated on a Waters BEH Glycan chromatography column, 100 × 2.1 mm i.d., 1.7 μm BEH particles, with 100 mM ammonium formate, pH 4.4, as solvent A and ACN as solvent B. The separation method used a linear gradient of 25–38% solvent A at a flow rate of 0.40 ml/min in a 27-min analytical run. Samples were maintained at 10°C before injection, and the separation temperature was 60°C. Data processing was performed using an automatic processing method with a traditional integration algorithm, after which each chromatogram was manually corrected to maintain the same intervals of integration for all the samples.

All obtained chromatograms were separated in the same manner into 24 peaks (84). Peaks contained distinctive N-glycans (Supplementary Figure 1). The amount of glycans in each peak was expressed as a percentage of the total integrated area. Derived traits were calculated according to the following formulas: for agalactosylated G0 = GP1 + GP2 + GP3 + GP4 + GP6, with one galactose G1 = GP7 + GP8 + GP9 + GP10 + GP11, with two galactoses G2 = GP12 + GP13 + GP14 + GP15, and sialylated glycans S = GP16 + GP17 + GP18 + GP19 + GP21 + GP22 + GP23 + GP24.

#### Cytokine Quantification With Multiplexed Luminex Analyses

Serum concentrations of cytokines, chemokines, and growth factors (listed in Supplementary Table 6) were analyzed using the 38-plexed Milliplex MAP Kit (cat.no. HCYTMAG-60K-PX38) according to the manufacturer's recommendations (Merck-Millipore Corp., Billerica, MA, USA). Analyses were performed in single reactions. Quantification of the markers was performed with a Bio-Plex 200 Luminex instrument and Bio-Plex Manager software (Bio-Rad, Sweden). Concentration of each marker was determined from an 8-point standard curve using five parameter logistic regression. Minimum detectable concentration (MinDC) was determined for each marker separately using the lowest concentration on the standard curve's linear phase [MinDC = c(low) + 2SD where SD is standard deviation]. The samples below MinDC were given a value of 50% of MinDC. In total, 38 different cytokines were isolated and quantified from 30 representative samples (*n* = 20 diet group, *n* = 10 control group) gathered from the study population.

#### Quality Control and Statistical Analysis of White Blood Cell Levels, IgG Glycome, and Cytokine Profile

Prior to statistical analysis, we checked the data for skewness, normality, and outliers with dot plots and histograms. Because generalized estimating equations (GEEs) allow non-Gaussian distributions, we did not apply further normalization. To reduce excess variance caused by outliers, measured outcomes were excluded from the analysis if the values exceeded ±4SD (IgG glycome, cytokines) or ±3SD (blood cell counts) from the mean.

#### Statistical Analysis of the Glycome, Circulating Number of WBCs, and the Cytokine Profile

GEE with a linear link and working independence correlation structure was used for the statistical analysis IgG glycome, circulating number of WBCs, and the cytokine profile. In the main analysis, we utilized the GEE to investigate whether IgG levels and IgG glycome composition, WBC counts, and cytokine levels differed in the diet and control group across any of the time points when accounting for between-subject variability and age. In total, samples from 42 subjects (diet group *n* = 25, control group *n* = 17) were included in the analysis. *P*-value adjustment for multiple testing was carried out using an FDR for all analyses conducted on glycome, and cytokine variables. The software used for statistical analysis was R (version 3.3.3 or higher, https://www.r-project.org).

### Transcriptome

#### Library Preparation, Sequencing, Read Alignment, and Batch Effect

Transcriptome was quantified in peripheral leukocytes extracted from blood samples. The sequencing RNA library of each sample was processed using Illumina TruSeq according to the protocol provided by the manufacturer (https://www.illumina.com). The utilized Illumina protocol was paired end and strand specific, and the applied read depth for library preparation was set to 2 × 100 bp. Sequencing of the RNA libraries was carried out with the Illumina HiSeq2000 sequencing platform.

#### Differential Expression Analysis

We further processed sequence alignments with the DESeq2 software (http://bioconductor.org/packages/DESeq2/) to assemble transcripts, quantify the expression levels, and analyze differentially expressed genes. DESeq2 applies its own normalization methods and independent filtering to raw read RNA-Seq data. Before statistical analysis, some of the low expressed genes were excluded from the analysis if they matched the criteria: (i) gene had zero read counts across samples and (ii) gene had lower than five read counts in at least five samples. We further excluded additional samples from the analysis according to three criteria: (i) prior information on incompetence to follow the study protocol, (ii) sample outliers based on Cook's distance, pairwise MA plots, and sample distance heat maps, and (iii) subjects without all three time-point measurements. After applying these exclusions, the final differential expression analysis set of samples included 111 samples from 37 participants (diet group *n* = 24, control group *n* = 13) included in the differential expression analysis.

#### Statistical Analysis of Transcriptome

To identify differentially expressed genes we used the likelihood ratio test to conduct a nested time-course study with DESeq2 (H_0_ = Group + Time + Group^*^Subject, H_1_ = Group + Time + Group^*^Subject + Group^*^Time). We investigated whether genes were differentially expressed between the diet and control group across any of the time points when accounting for the between-subject variability. *Post-hoc* analysis of the Likelihood ratio test was also conducted for the diet and control group only (H_0_ = Subject, H_1_ = Subject + Time) to further explore within-group changes. In addition, Wald tests were applied within the DESeq2 interface for testing contrasts for the between/within group comparison across any two individual time points. A *q*-value of 0.05 for FDR was used to adjust for multiple testing.

#### Pathway Analysis of Gene Level Data

Downstream pathway analysis was conducted to identify enriched and overrepresented biological pathways. A web-based tool ConsensusPathDB-human database (http://cpdb.molgen.mpg.de) was used for analysis as it combines a wide set of integrated databases. Enrichment and overrepresentation analysis focused on determining pathways from the Reactome and Kyoto Encyclopedia of Genes and Genomes databases.

## Code Availability

The bioinformatics scripts/codes generated for statistical analysis purposes during the current study are available from the corresponding author on reasonable request.

## Data Availability

Limited data generated from the current study are available for third party investigators. To gain access to the current study data, investigators will need to apply and agree to by signature the necessary requirements and terms of data distribution agreements set forth by the National Institute for Health Welfare, Helsinki, Finland.

## Ethics Statement

This study was carried out in accordance with the recommendations of Ethical Committee at the University of Jyväskylä with written informed consent from all subjects. All subjects gave written informed consent in accordance with the Declaration of Helsinki. The protocol was approved by the Ethical Committee at the University of Jyväskylä.

## Author Contributions

HS was responsible for the analysis and the majority of writing. MP, KK, and JH supervised the project and contributed significantly to the writing and editing of the manuscript. JL, JT, and ZJ contributed to the statistical analysis and editing of the manuscript. JJH, VI, JA, KH, and MP designed the original study and were responsible for conducting the trial. Other authors contributed to analysis and interpretation of the results based on their expertise (IG, JJ, GL—glycomics; JI—exercise immunology; JH, AV—cytokines). All the authors accepted the final version of the manuscript.

### Conflict of Interest Statement

GL is founder and CEO of Genos—a private research organization that specializes in high-throughput glycomic analysis and has several patents in this field. IG and JJ are employees of Genos. The remaining authors declare that the research was conducted in the absence of any commercial or financial relationships that could be construed as a potential conflict of interest.

## Abbreviations

BCR: B-cell receptor
FceRI: Fc epsilon receptor-like protein
FCRL: Fc receptor-like protein
FDR: false discovery rate
GEE: generalized estimating equation
HSC: hematopoietic stem cell
IgE: immunoglobulin E
IgG: immunoglobulin G
IP10: interferon-gamma induced protein 10
METh/wk: metabolic equivalent hours per week
MHC: major histocompatibility complex
MinDC: minimum detectable concentration
ROS: reactive oxygen species
sCD40L: soluble CD40 ligand
S-hs-CRP: serum high-sensitivity C-reactive protein
T_FH_: T-follicular helper
T_H_: T-helper
TNF-α: tumor necrosis factor alpha
WBC: white blood cell.
