# Supplementary material for: Molecular Pathways Mediating Immunosuppression in Response to Prolonged Intensive Physical Training, Low-Energy Availability, and Intensive Weight Loss
Source: Front Immunol. 2019 May 3;10:907. doi: 10.3389/fimmu.2019.00907 (PMC6511813; doi:10.3389/fimmu.2019.00907)
Supplement: Supplementary file 2 [file Data_Sheet_1.PDF]

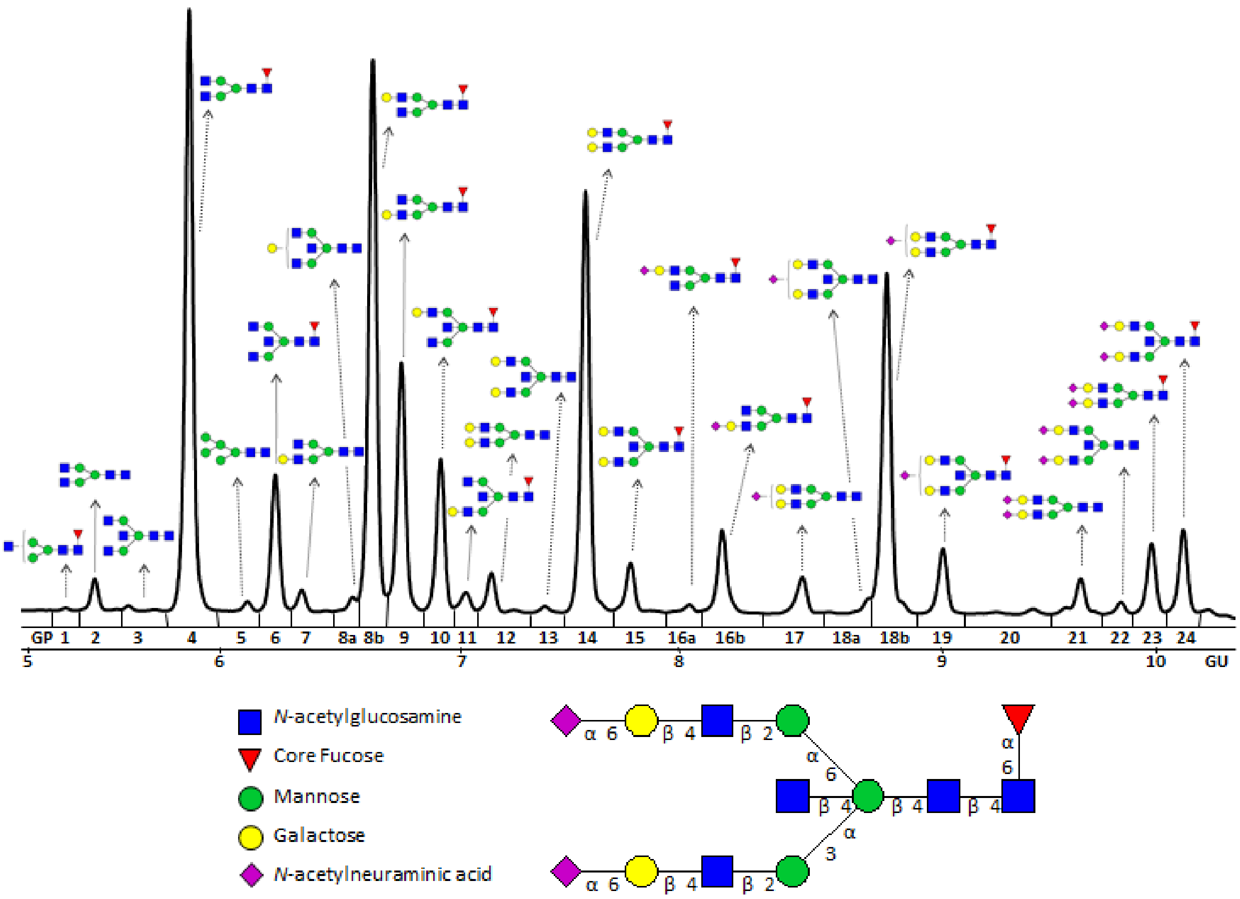

Supplementary figure 1. Representative chromatogram of 2-AB labeled N-linked glycans released from IgGs isolated from human plasma and separated by HILIC-UPLC. The integration areas, together with a major structure presented in each glycan peak are given. Glycan peaks are numbered from GP1-GP24, as used in the paper.
